# No Moderating Effect of 5-HTTLPR on Associations Between Antenatal Anxiety and Infant Behavior

**DOI:** 10.1016/j.jaac.2013.02.010

**Published:** 2013-05

**Authors:** Elizabeth C. Braithwaite, Paul G. Ramchandani, Thomas G. O'Connor, Marinus H. van IJzendoorn, Marian J. Bakermans-Kranenburg, Vivette Glover, Elena Netsi, Jonathan Evans, Michael J. Meaney, Susannah E. Murphy

**Affiliations:** a University of Oxford; b Imperial College London; c University of Rochester Medical Center; d Leiden University; e University of Bristol; f McGill University

**Keywords:** 5-HTTLPR, antenatal anxiety, gene-by-environment interaction (G×E), fetal programming, serotonin transporter

## Abstract

**Objective:**

Maternal antenatal anxiety is associated with an increased risk of behavioral disturbances in offspring. Recent work has suggested that the effect of maternal antenatal anxiety on infant temperament at 6 months is moderated by the serotonin transporter polymorphism 5-HTTLPR, with carriers of the short allele more susceptible to the adverse behavioral outcomes of maternal antenatal anxiety. These findings, however, are yet to be replicated and extended beyond infancy. The aim of the current study was to assess this same potential moderator (5-HTTLPR) in a large population-based cohort study, and to determine whether or not the effects persist into childhood and early adolescence.

**Method:**

Data from the Avon Longitudinal Study of Children and Parents (ALSPAC) cohort (N = 3,946) were used to assess whether the 5-HTTLPR genotype moderated the association between self-reported maternal antenatal anxiety (Crown Crisp Index) in pregnancy, and child temperament at 6 months (Infant Temperament Questionnaire), and also later behavioral and emotional problems on the Strengths and Difficulties Questionnaire from age 4 to 13 years.

**Results:**

We found no evidence to suggest that the 5-HTTLPR polymorphism moderated the effects of maternal antenatal anxiety on infant temperament at 6 months or infant behavioral and emotional problems from childhood through to adolescence.

**Conclusion:**

Our results, based on a large prospective community sample that assessed children from infancy to early adolescence, provide a thorough test of, but no evidence for, a genetic moderation of the effects of maternal antenatal anxiety by 5-HTTLPR.

There is a growing awareness that environmental exposure to stress can shape developmental trajectories as early as the fetal period. This has been described in terms of the fetal programming hypothesis^1,2^ that stipulates that the phenotype of a fetus may be altered during the antenatal period in accordance with maternal cues. Maternal anxiety during pregnancy is associated with an increased risk of behavioral and emotional disturbances in offspring.^3-5^ A prominent challenge in this field is to disentangle the extent to which intergenerational transmission of mood disturbances are attributable to genetic or environmental factors, or both. The recent use of genetically sensitive study designs have confirmed that stressful insults that influence the antenatal environment, such as high levels of maternal anxiety, do have long-term effects on offspring outcomes that are, in part, independent of genetic factors.^6^

It is clear, however, that there are individual differences in the extent to which developing fetuses are susceptible to maternal antenatal anxiety, and developmental programming more broadly. Early investigations in this area were interpreted largely in light of the classic “diathesis-stress model”,^7^ which proposed that some children are more susceptible to developing behavioral and emotional problems in unfavorable environments than are others. Supporting evidence indicates, for example, that psychosocial stress, as one type of unfavorable environment, predicts behavioral and emotional problems in children depending on individual differences in phenotypic factors, such as temperament^8-10^ and impulsivity.^11^ Genetic factors also define susceptibility to environmental influences. For example, the serotonin transporter polymorphism, 5-HTTLPR, has been identified as a moderator of the impact of stressful life events on depressive symptoms,^12^ whereby the short (s) allele is thought to confer susceptibility to depressive symptoms. However, there is considerable debate about the validity of these findings, given two negative meta-analyses of the gene-by-environment (G×E) interactions,^13,14^ one positive meta-analysis, and two relevant papers that suggest that the initial finding may be a false-positive result.^15,16^

We extend this line of research in the current study by testing the hypothesis that 5-HTTLPR moderates the effect of maternal antenatal anxiety on infant temperament and behavioral and emotional problems. Two previous investigations have addressed this question.^17,18^ In a small-scale study of 75 mothers and their children, Oberlander *et al.* (2010) reported that high levels of maternal antenatal anxiety predicted anxious and depressive symptoms in children, but only those with 2 copies of the s allele. In contrast, increased aggression and externalizing behaviors were predicted by high third-trimester anxiety only in children with 2 copies of the l allele. These findings suggest that 5-HTTLPR does moderate behavioral outcomes of maternal antenatal anxiety; however, these results must be interpreted with caution, given the small sample size. In a larger cohort study (N = 1,513), Pluess *et al.*^17^ reported that the 5-HTTLPR polymorphism significantly moderated the effect of second trimester maternal antenatal anxiety on infant negative emotionality. They found that maternal reports of emotional difficulties at 6 months were predicted by maternal self-reported antenatal anxiety only in infants who carried at least one copy of the s allele, whereas there was no association in those infants homozygous for the l allele. Such evidence is consistent with the idea that 5-HTTLPR may moderate the effect of maternal antenatal anxiety on the developing fetus, with s allele carriers showing increased susceptibility to such an environmental insult.

The aim of the current study is to provide a substantive replication of these findings by assessing the moderating effect of 5-HTTLPR on the association between maternal antenatal anxiety and temperament in infants 6 months of age, in a large population cohort, and to extend the previous findings by examining whether these effects persist beyond infancy.

## Method

### Participants

The present study is an analysis of data collected as part of the Avon Longitudinal Study of Parents and Children (ALSPAC).^19^ ALSPAC is a large, population-based, longitudinal study in which pregnant women from the Avon area in the United Kingdom were recruited, with delivery dates between April 1991 and December 1992. Initially, 14,551 pregnant women were enrolled during the early stages of the study, and 13,801 mothers remained within the study. There were a total of 14,062 live births, and 13,985 surviving offspring at 12 months. Questionnaires were sent to parents at regular time intervals during pregnancy and after birth. Following childbirth, DNA samples were obtained from more than 10,000 children, and 5-HTTLPR genotypes were available for 5,631 individuals. Our analyses were carried out only using data from infants of white ethnicity, because 5-HTTLPR allele frequencies are inconsistent between different ethnic groups.^20^ Thus, a subsample of n = 5,084 infants was used for the current analysis; however, sample size for analyses predicting behavioral outcomes from genetic data and prenatal anxiety was somewhat lower because of missing data on covariates (numbers [n] ranged from 3,946 to 4,087). Table 1 presents the demographic characteristics of the subsample used for analysis. Ethical approval for the study was obtained from the ALSPAC law and ethics committee, and from local research and ethics committees. All participants provided informed consent.

### Procedures and Measures

#### Maternal Antenatal Anxiety

At 18 and 32 weeks of pregnancy, maternal anxiety was assessed using the anxiety items from the Crown Crisp Index, a validated self-rating inventory.^21,22^ The Crown Crisp Index is a 24-item questionnaire with 4 subscales each composed of 8 items. Participants are asked to rate their responses to questions relating to feelings and behaviors on a 4-point scale from “very often” to “never.” Studies using this measure have previously demonstrated a robust association between maternal antenatal anxiety and adverse child behavior outcomes.^3^

#### Infant Temperament at 6 Months

The Infant Temperament Questionnaire^23^ was used to assess child temperament at 6 months of age. The questionnaire identifies 9 domains of temperament: activity, adaptability, approach, distractibility, intensity, mood, persistence, rhythmicity, and threshold. Parents completed the questionnaire using a 6-point scale response from “almost never” to “almost always.” This questionnaire has previously verified test reliability and internal consistency.^23^ One temperament characteristic (infant reactivity) was chosen a priori for the present study. Infant reactivity was chosen for assessment as it has been shown to be an important characteristic in terms of infant plasticity and is influenced by maternal antenatal anxiety.^24^ Individual items from the Infant Temperament Questionnaire corresponding to reactivity were identified independently by three of the authors (P.R., M.B.-K., and M.vIJ.). The identified items fell almost exclusively within the adaptability, approach, intensity and threshold subscales. Therefore, a scale to assess infant reactivity was established by calculating the sum of the z scores for the adaptability, approach, intensity, and threshold domains, and this showed good internal consistency (α = 0.768). A similar method has been used previously to calculate an infant reactivity measure.^25^ The questions in these scales show significant overlap with those used in the study by Pluess *et al.*^17^ For example, there are similar questions about the infant's reaction to loud noises, the degree of protest when being dressed, and how quickly the infant calms down after an unpleasant stimulus.

#### Child Emotional and Behavioral Problems

The Strengths and Difficulties Questionnaire (SDQ) is a well-validated parent-report assessment of child emotional and behavioral problems.^26^ The SDQ assesses specific child symptoms related to attention/hyperactivity, conduct, and emotional problems. In addition, the total SDQ score shows good predictive validity of clinician-rated mental health disorders.^27^ Mothers completed the SDQ when their child was 4, 7, 9, 11.5, and 13 years of age.

#### Genotyping

Child DNA was extracted from peripheral blood samples and genotyped as described previously.^28^ The 5-HTTLPR allele has been shown to be triallelic: a single nucleotide substitution (A>G) within the l allele results in a functional AP2 transcription factor binding site that inhibits transcription, creating an allele functionally equivalent to the s allele.^29^ Thus, with regard to protein expression level, the L_G_ allele can be functionally grouped with the s allele. For analysis, the six genotypes were grouped by expression level: low expression: SS, SL_G_, L_G_L_G_; medium expression: SL_A_, L_G_L_A_; and high expression: L_A_L_A_. The triallelic 5-HTTLPR variable was treated as a polytomous variable, and entered into the statistical models (as described below) using “low,” “medium,” and “high” group terms.

### Analysis Plan

The analysis was undertaken using the Statistical Package for the Social Sciences (SPSS). We considered child gender as a potential moderator in the analyses. We ran two separate models. The first model examined a maternal antenatal anxiety (at 18 or 32 weeks' gestation) × 5-HTTLPR interaction to predict temperament at 6 months. A linear regression model was constructed with infant reactivity as the outcome measure. At step 1, we entered possible confounders: maternal age, smoking and alcohol consumption during pregnancy, maternal education, household crowding (an index of the number of residents in a dwelling divided by the number of rooms in the dwelling), maternal education, gender, living with partner, and maternal postnatal depression. At step 2, we entered the main predictor variables, 5-HTTLPR genotype and maternal antenatal anxiety at 18 weeks. At step 3, three two-way interaction terms were created and entered into the model: maternal anxiety × 5-HTTLPR, maternal anxiety × gender and gender × 5-HTTLPR. All interaction terms were created using centred variables to avoid problems of multicollinearity, and there was no correlation between the centered genotype and maternal antenatal anxiety variables (r = 0.003, *p* = 0.832). Finally, at step 3, we created a three-way interaction term (maternal anxiety × 5-HTTLPR × gender), and entered this into the model. The model was then reconstructed, with maternal antenatal anxiety at 18 weeks replaced by maternal antenatal anxiety at 32 weeks.

The second model predicted behavioral problems on the SDQ from age 4 to 13 years using the emotional, conduct, and hyperactivity subscales as well as the total problem scale. Generalized estimating equations were used to generate parameter estimates (and standard errors) because this method accounts for the nested structure of the data (one to five SDQ measurements were available for each child). In addition, the resulting covariation among the behavioral problem scales varied from modest to large (discussed below), and this is managed within a generalized estimating equations framework.^30^

## Results

Compared to the entire ALSPAC sample, the subsample of infants used for analysis were generally at lower risk, indexed, for example, by older maternal age, higher maternal educational qualifications, and greater likelihood of living in a two-parent family. In addition, they had mothers who reported lower antenatal anxiety and depression and postnatal depression. They also showed comparatively less reactivity at 6 months and fewer behavioral and emotional problems on the SDQ. The effect sizes of these differences were generally small, but were significant because of the large sample size. For example, for emotional problems on the SDQ, effect size differences ranged from 0.02 to 0.09 of a standard deviation.

### Infant Reactivity at 6 Months

Previous findings have shown that maternal antenatal anxiety predicts infant behavioral outcomes, using the SDQ as a childhood measure of emotion and behavior; here we found that maternal antenatal anxiety also predicts infant temperament at 6 months. That is, the infants of mothers who had high levels of anxiety during pregnancy had a more reactive temperament than those infants of mothers who had average anxiety levels (*p* < 0.001). In line with previous findings, we found a significant effect of child gender to predict infant temperament at 6 months (*p* < 0.001), such that boys have a less reactive temperament than girls. We did not, however, find a significant effect of the 5-HTTLPR genotype alone or an antenatal anxiety × 5-HTTLPR genotype interaction to predict infant temperament at 6 months. We did find a gender × 5-HTTLPR interaction (*p* = 0.038), which significantly predicted infant temperament at 6 months. Further exploration of this interaction revealed that for female infants only, the 5-HTTLPR genotype predicted reactivity: those females with a high expression level of the serotonin transporter had a significantly more reactive temperament at 6 months than females with a low serotonin transporter expression level (*p* < 0.05). Results of the linear regression model are shown in Table 2.

We re-ran this model with maternal antenatal anxiety at 32 weeks as the independent variable and found similar results: main effects of maternal antenatal anxiety and child gender to predict temperament, but no main effect of 5-HTTLPR or a 5-HTTLPR × maternal anxiety interaction. Furthermore, because of the interest in maternal antenatal depression as a possible predictor of child temperament and behavior problems, we reconstructed the model using maternal antenatal depression at 18 and 32 weeks, child gender, and 5-HTTLPR as predictors of temperament. Our results remained consistent, with significant effects of child gender and maternal antenatal depression, but no main effect of 5-HTTLPR or a 5-HTTLPR × antenatal depression interaction.

For equitable comparison with previous research, the 5-HTTLPR genotype data was recoded into the biallelic genotypes (ss, sl, and ll) and re-entered into the regression model in place of the triallelic 5-HTTLPR genotype variable. As with the triallelic coding of the serotonin genotype, we found no evidence of a significant maternal antenatal anxiety-by-genotype interaction to predict infant reactivity at 6 months.

### Behavioral Problems, Age 4 to 13 Years

Stability of individual differences varied from age 4 to 13 years; for the total problem scale, the correlations ranged from r = 0.44 (age 4 years and age 13 years) to r = 0.74 (age 11.5 years and age 13 years).

Given the stability of individual scores, analyses of the SDQ data were based on generalized estimating equations, which account for the dependence within individuals across time. The first, most basic prediction model included the five measures of SDQ as the outcome and 5-HTTLPR genotype, maternal antenatal anxiety at 18 weeks, and child gender as predictors; interaction terms were created for 5-HTTLPR × antenatal anxiety, 5-HTTLPR × child gender, and the three-way interaction among 5-HTTLPR genotype, maternal antenatal anxiety, and child gender. Results for the total problem scale indicated that higher scores were associated with male gender (B 1.04 [SE 0.39], *p* < .01) and maternal antenatal anxiety (B 0.33 [SE 0.05], *p* < .001), but no evidence of a significant main effect of 5-HTTLPR (*p* = 0.91) or an antenatal anxiety × 5-HTTLPR interaction (*p* = 0.54). Analyses of the symptom subscales also indicated a lack of significant prediction from 5-HTTLPR (*p* values ranging from 0.71 to 0.99) and no evidence of a maternal antenatal anxiety × 5-HTTLPR interaction (*p* values ranged from .18 to .79). There was consistent evidence for maternal antenatal anxiety (for conduct problems, 0.06 [SE 0.01], *p* < .001; for infant emotional problems, B 0.09 [SE 0.02], *p* < .001; for hyperactivity, B 0.14 [SE 0.02], *p* < .001); child gender was significant for hyperactivity (B 0.92 [SE 0.19], *p* > 001); there was no evidence for prenatal anxiety × child gender interactions (*p* values ranged from .47 to .93). Analyses using maternal antenatal anxiety at 32 weeks gestation yielded parallel findings. Furthermore, we also re-analyzed the data using the biallelic coding of the 5-HTTLPR genotype and obtained comparable results. Finally, we re-analyzed the data substituting antenatal maternal depression for antenatal anxiety and found consistent results: significant main effects for child gender and maternal antenatal depression but no evidence of a significant main effect for 5-HTTLPR or a 5-HTTLPR × maternal antenatal depression interaction.

## Discussion

We conducted analyses of a large community of sample of children for whom we had data from the prenatal period to age 13 years to examine the role of 5-HTTLPR on behavioral outcomes from infancy to adolescence, and particularly the degree to which this gene moderated the impact of maternal prenatal anxiety on child outcomes. We obtained one positive finding: in girls only, the serotonin transporter genotype predicted reactive temperament at 6 months. Specifically, girls with a high expression level of the serotonin transporter had a more reactive temperament than those with low expression level. That a single polymorphism may contribute to a behavioral characteristic such a temperament is intriguing and requires further exploration. However, we are cautious about the interpretation of this finding, as it may represent a false-positive result, given the number of statistical tests carried out. It therefore requires replication in another large cohort before conclusions regarding this association can be drawn.

On the other hand, we obtained many null findings, despite the large sample size, long-term period of study, and detailed symptom measures. The main finding of this study is that the effect of antenatal anxiety during the second and third trimester on infant temperament at 6 months and later behavioral problems is not moderated by the 5-HTTLPR genotype. Although our results show that maternal antenatal anxiety predicts infant temperament and later behavioral problems, consistent with previous findings,^3,17^ we found no evidence for a gene-by-environment interaction between maternal antenatal anxiety and 5-HTTLPR to predict infant temperament or behavioral outcomes. These results remained robust when a three-way interaction to include child gender was entered into the model.

These findings are not consistent with those of Pluess *et al.* (2011), which suggested that a combination of two short alleles and high levels of maternal antenatal anxiety present a cumulative risk factor for infant emotional difficulties at 6 months of age, in line with the diathesis-stress/dual-risk model. In the current study, however, we found no evidence of any type of interaction that resembled either a diathesis-stress or differential susceptibility model. In an attempt to comprehensively replicate previous methodology, biallelic re-coding of the 5-HTTLPR genotype did not significantly change the outcome of the regression model; the biallelic genotype did not moderate the effects of antenatal anxiety on infant temperament. The variation in findings between the two studies may be attributable to a number of factors, including the use of different questionnaire measures. As a measure of infant temperament, the present study used infant reactivity as assessed by the Infant Temperament Questionnaire, whereas Pluess *et al.* used a revised version of the Infant Behavior Questionnaire, with the negative emotionality subscale for analysis. A different measure of maternal antenatal anxiety was also used: the anxiety scale of the Crown Crisp Index and the Brief Symptom Inventory. However, both are validated self-report measures of anxiety, and both have been used extensively, and are therefore unlikely to be the source of discrepancy.

It is possible that other methodological differences between the current study and the Pluess *et al.* paper may underlie the differences in results. However, that two large cohort studies resulted in very different outcomes suggests that the role of the serotonin transporter polymorphism in moderating antenatal environmental influences on offspring behavioral outcomes may not be straightforward and consistent in differing circumstances. There is some evidence from studies of adults that the serotonin transporter polymorphism moderates the response to stressful events during the childhood years. However, even here the findings are not consistent.^13,31,32^ Findings relating to childhood outcomes are even less consistent,^28,33^ and our results suggest that this moderation effect may not extend to the antenatal period. It should be noted, however, that replication of gene-by-environment (G×E) findings may be critically dependent on the quality of the assessment of the environment in the G×E equation. Both in the original Pluess *et al.* study as well as in our study on antenatal influences, the environment was assessed with a short self-report questionnaire. A more sophisticated observational or interview procedure might have decreased the error component in E, making it easier to replicate the G×E outcome with smaller samples. An even better way to test for G×E effects would be to experimentally manipulate the environment. In genetically informed randomized controlled trials, randomization prevents hidden moderator effects on the environment and guarantees the independence of moderator and outcome, while the environment is manipulated and assessed in standard ways.^34,35^

There are a number of strengths to this study. First, the participants were drawn from a large population-based cohort; thus this study is relatively free of selection biases, which are usually associated with experimental and clinical trials. Second, the measures used in this study, namely, the Crown Crisp Index, Infant Temperament Questionnaire, and the SDQ, have been extensively used and are well validated. Third, the sample size is very large and the largest to date used to investigate the effects of the serotonin transporter polymorphism and antenatal mood disturbance. Therefore our study had sufficient statistical power to detect all but very small effects. Pluess *et al.* (2011) found an interaction effect size of f2 = 0.004, and a power analysis revealed that to find this effect size with 80% power, a sample size of 2,412 participants is required. Therefore, with a sample size of 1,513, their study was underpowered, whereas our study, with a sample of 3,943 participants, was sufficiently powered to find such an effect. As highlighted by Duncan and Keller (2011), many G×E studies are significantly underpowered, resulting in a situation in which positive findings from such studies may actually represent type 1 errors. Furthermore, given that multiple candidate gene by environment studies are carried out within and across many laboratories, it is inevitable that *p* values of less than .05 are found. Preferential publication of positive results means that false-positive results enter the literature, and are thus subsequently difficult to replicate. Notably, there is a strong precedent for this in the candidate gene literature.^36^ If this is the case, then even well-powered replication attempts, such as the one reported here, will fail to replicate previous findings. Duncan and Keller are correct to conclude that well-powered direct replications deserve more attention than novel G×E studies and indirect replications. We believe that this study is one such direct, well-powered replication attempt.

However, this study also has limitations, which should be considered. First, measures of infant behavior were based solely on maternal reports. Although this is perhaps inevitable in a large cohort study, it nonetheless raises the potential for the ratings of infant behavior to be subject to reporter bias. That is, anxious mothers may be more likely to over- or mis-report behavioral disturbances of the infant, which may have led to greater associations between the variables. However, this limitation should also apply to the previous studies in the field, and cannot alone explain the discrepancy in findings. Second, there is evidence that the subsample used for analysis differed from the entire sample. This mainly related to the demographic characteristics of the mothers of the children; for example, they generally had lower levels of mood disturbance and higher levels of educational qualifications. Therefore it is possible that the subsample used for analysis was biased and did not generalize to the entire population.

In conclusion, our study has shown that, within a large population-based cohort in England, the effects of maternal antenatal anxiety on infant behavioral outcomes at 6 months and up to 13 years are not moderated by the serotonin transporter genotype. This study is a well-powered replication attempt; however our findings are inconsistent with previous work. It is therefore likely that the role of 5-HTTLPR in moderating the effects of fetal programming, if any, is more subtle and variable than previously thought.

## Figures and Tables

**Table 1:** Demographic Characteristics of the Sample

Variable	Whole ALSPAC Sample (N = 13,801)	Subsample (n = 5,084)
Maternal age (y) at birth, mean, SD	28.0, 4.97	29.14, 4.60
Mother's highest educational qualification, n (%)		
CSE	2,522 (20.2)	671 (13.2)
Vocational	1,228 (9.8)	441 (8.7)
0 level	4,323 (34.6)	1,800 (35.5)
A level	2,803 (22.5)	1,324 (26.1)
Degree	1,607 (11.0)	833 (16.4)
Living situation (during pregnancy), n (%)		
Living with partner	12,426 (84.7)	4,766 (94.8)
Living without partner	1,160 (8.5)	262 (5.2)
Smoking during pregnancy, n (%)	2,436 (20)	741 (16.9)
Alcohol during pregnancy, n (%)	2,200 (31.8)	1,052 (20.7)
Maternal anxiety during pregnancy (18 weeks), mean, SD	4.93, 3.55	4.63, 3.36
Maternal depression during pregnancy (18 weeks), mean, SD	6.98, 4.86	6.45, 4.56
Maternal anxiety during pregnancy (32 weeks), mean, SD	5.14, 3.60	4.87, 3.45
Maternal depression during pregnancy (32 weeks), mean, SD	7.08, 5.08	6.71, 4.86
Maternal postnatal anxiety (8 weeks), mean, SD	3.42, 3.34	3.32, 3.22
Maternal postnatal depression (8 weeks), mean, SD	6.06, 4.80	5.85, 4.59
Gender, n (%)		
Male	7,318 (51.7)	2,685 (52.8)
Female	6,825 (48.3)	2,399 (47.2)
Child 5HTTLRP genotype, n (%)		
Low expression	—	1,187 (23.3)
Medium expression	—	2,595 (51.1)
High expression	—	1,302 (25.6)
Child reactivity score at 6 months, mean, SD		
Males	−0.15, 2.69	−0.26, 2.69
Females	0.16, 2.76	0.07, 2.77

Note: ALSPAC = Avon Longitudinal Study of Parents and Children; CSE = Committee on Special Education.

**Table 2:** Summary of Linear Regression Analysis for Antenatal Anxiety and 5-HTTLPR Genotype as Predictors of Infant Reactivity at 6 Months

Predictor variables	Antenatal anxiety at 18 weeks				Antenatal anxiety at 32 weeks			
B	β	t	*p*	B	β	t	*p*
Step 1								
Maternal age	−0.014	−0.023	−1.376	.172	−0.019	−0.031	−1.847	.065
Smoking during pregnancy	−0.048	−0.011	−0.635	.526	−0.013	−0.003	−0.178	.859
Alcohol during pregnancy	−0.008	−0.009	−0.553	.581	−0.002	−0.002	−0.12	.904
Maternal education	0.004	0.002	0.098	.922	0.008	0.004	0.215	.830
Household crowding	0.211	0.045	2.716	.007	0.197	0.043	2.625	.009
Gender	0.328	0.060	3.719	.000	0.312	0.057	3.610	.000
Living with partner	−0.366	−0.028	−1.720	.086	−0.46	−0.036	−2.275	.023
Postnatal depression	0.051	0.084	4.246	.000	0.059	0.098	5.272	.000
Step 2								
Antenatal anxiety	0.174	0.211	4.151	.000	0.164	0.202	4.091	.000
5-HTTLPR genotype	−0.300	−0.077	−1.532	.126	−0.335	−0.086	−1.747	.081
Step 3								
Antenatal anxiety × 5-HTTLPR genotype	−0.050	−0.042	−0.828	.408	0.028	0.024	0.491	.624
5-HTTLPR genotype × gender	0.262	0.105	2.076	.038	0.293	0.117	2.374	.018
Gender × antenatal anxiety	−0.056	−0.106	−2.103	.036	−0.073	−0.141	−2.885	.004
Step 4								
Antenatal anxiety × 5-HTTLPR genotype × Gender	0.030	0.040	0.787	.431	0.003	0.004	0.077	.939

Note: N = 3,946, derived from those participants with genetic, prenatal anxiety, and temperament data at 6 months.
